# Hypersensitivity in Borderline Personality Disorder during Mindreading

**DOI:** 10.1371/journal.pone.0041650

**Published:** 2012-08-03

**Authors:** Carina Frick, Simone Lang, Boris Kotchoubey, Simkje Sieswerda, Ramona Dinu-Biringer, Moritz Berger, Sandra Veser, Marco Essig, Sven Barnow

**Affiliations:** 1 Department of Clinical Psychology and Psychotherapy, University of Heidelberg, Heidelberg, Germany; 2 Institute of Medical Psychology and Behavioral Neurobiology, University of Tuebingen, Tuebingen, Germany; 3 German Cancer Research Center (DKFZ), Heidelberg, Germany; 4 Department of Neuroradiology, University of Erlangen, Erlangen, Germany; The University of Melbourne, Australia

## Abstract

**Background:**

One of the core symptoms of borderline personality disorder (BPD) is the instability in interpersonal relationships. This might be related to existent differences in mindreading between BPD patients and healthy individuals**.**

**Methods:**

We examined the behavioural and neurophysiological (fMRI) responses of BPD patients and healthy controls (HC) during performance of the ‘Reading the Mind in the Eyes’ test (RMET).

**Results:**

Mental state discrimination was significantly better and faster for affective eye gazes in BPD patients than in HC. At the neurophysiological level, this was manifested in a stronger activation of the amygdala and greater activity of the medial frontal gyrus, the left temporal pole and the middle temporal gyrus during affective eye gazes. In contrast, HC subjects showed a greater activation in the insula and the superior temporal gyri.

**Conclusion:**

These findings indicate that BPD patients are highly vigilant to social stimuli, maybe because they resonate intuitively with mental states of others.

## Introduction

The awareness that other people have a mental state different from our own is referred to as “theory of mind (ToM)” [1] or “mentalizing” [2]. Mentalizing incorporates inferring a mental and emotional state from multiple sources, including non-verbal cues, such as facial expressions and gaze direction, as well as knowledge about the other person’s perspective and beliefs [3]. The ability to mentalize constitutes a central aspect of social cognition, which is regarded to be a human-specific skill that forms a crucial prerequisite to function in social groups [4], [5].

Borderline personality disorder (BPD) is a severe mental disorder, which is characterized by disturbance in interpersonal relations and emotional dysregulation, particularly within social contexts [6]–[8]. It has been proposed that differences in mentalizing between BPD and controls can lie at the bottom of this disturbance. However, a controversy exists regarding the direction of these differences. Whereas several authors assume that BPD patients have impaired mentalizing ability (e.g., [9], [10]), the ‘paradox’ hypothesis [11] maintains in contrast, that individuals with BPD exhibit enhanced mental state discrimination in the context of their impaired interpersonal relationships.

Although the relational style of BPD has been suggested to be the best discriminator for diagnosis [12], disturbed social relatedness in BPD has been explored only in a limited number of studies to date. In search for the causes underlying interpersonal dysfunction in BPD, most of the studies focused on facial emotion recognition using basic emotions. However, the results are highly inconsistent. Some studies showed generally impaired accuracy of BPD patients in emotion recognition [13], [14], others indicated a negativity bias [15], [16], still others found no alterations in emotion recognition (e.g., [17]], or even increased sensitivity for the detection of negative emotions [18]. The last result is in accord with the ‘paradox’ hypothesis described above. Similarly inconsistent were the results of neuroimaging studies investigating the activity of the amygdala as a correlate of emotional responses to facial emotion expression: some of them found increased amygdalar activity in BPD patients compared to healthy subjects [15], [19], other did not [20]. A reason for the controversial results might be, among others, the uncontrolled effect of comorbide factors (such as depression) in some BPD samples.

In the present study, we used the ‘Reading the Mind in the Eyes’ task (RMET) to assess subtle affective mentalizing abilities in BPD patients [21]. In the RMET, subjects have to attribute a mental state based on information derived from pictures portraying the eye region of the face. According to Baron-Cohen et al. [21], the RMET involves the first stage of mental state attribution, the “unconscious, rapid and automatic” decoding of the “language” of the eyes, without the subsequent (more cognitive) deduction of a mental content. Previous studies have shown that the eyes alone are a crucial feature of social and emotional processing [22], [23].

Neuropsychological studies showed that the processing in the RMET involves the medial prefrontal cortex (PFC), the orbitofrontal cortex, the amygdala, the superior temporal gyrus, and the temporal poles [24], [25]. In addition, lesion studies found impaired mental state discrimination in patients with damage of the amygdala, orbitofrontal cortex and medial PFC [26], [27]. The finding of amygdala activation is of particular interest in the light of the literature suggesting amygdala dysfunction in BPD [28], [29].

In line with the paradox hypothesis, a recent study by Fertuck et al. [30] reported enhanced ability in mental state discrimination in BPD patients as compared to healthy subjects using the RMET. However, one limitation of this study is that it remained unclear whether or not the results were borderline-specific or at least partially modulated by depression. Likewise, Scott et al. [31] found superior affective mentalizing abilities in healthy non-clinical persons with high (compared to low) borderline features. In another study by Preißler et al. [32], no group differences in the RMET were found between BPD patients and healthy subjects. In that study, however, severity of depressive symptoms was not controlled for. In addition, none of these studies have assessed the neural underpinnings of RMET in BPD patients.

The aim of the present study was to investigate neural circuits of affective mental state discrimination in BPD patients using the RMET. Based on the divergent findings of previous studies, we expected to find one of two possible patterns in the data: either patients with BPD would show enhanced mental state accuracy compared to healthy subjects, or no difference in mentalizing between the BPD and control groups would be found. Because the RMET involves amygdala activation and BPD patients show hyperactive amygdala responses, we expected BPD patients to exhibit hyperactivity in the amygdala relative to healthy subjects. Additionally, we aimed to evaluate the impact of depressive severity on RMET in patients with BPD.

## Materials and Methods

### Participants

The study group consisted of 21 non-medicated right-handed female BPD patients and 20 female healthy volunteers recruited from general population with advertisements in local newspapers and postings. All BPD patients had trauma history and fulfilled at least five of nine DSM-IV-TR criteria for BPD and did not have a history of schizophrenia-spectrum psychosis, bipolar type I affective disorder or current substance abuse during the previous six months. Exclusion criteria for healthy subjects comprised of any current or past DSM-IV Axis I or Axis II disorder. Further, exclusion criteria for all subjects were the history of head trauma, neurological diseases or any chronic illness as well as any contraindication to functional magnetic resonance imaging (fMRI), pregnancy and current suicidal thoughts.

All participants were informed about the aims and risks of this study and gave written informed consent. The study was approved by the Research Ethics Board of the University of Heidelberg in accordance with the declaration of Helsinki.

### Diagnostic and Clinical Measurements

The German version of the Structured Clinical Interview for DSM-IV Axis I Disorders (SCID-I) [33] was used to asses Axis I comorbidity including PTSD. Axis II diagnoses were determined using the German version of the Structured Clinical Interview for DSM-IV Axis II disorders (SCID-II) [34]. Affective instability was assessed with the German version of the Affective Lability Scale (ALS) [35], a self-report instrument shown to correlate with clinician-rated affective instability in patients with BPD [36]. Depression was documented with the German version of the 21-item Beck Depression Inventory (BDI) [37] and handedness with the Edinburgh Handedness Inventory (EHI) [38].

### Subject Characteristics

The BPD and HC groups did not differ in age, education level, or handedness (Table 1). The BPD patients scored higher in depression and frequency of symptoms of affect liability. Axis I and II comorbidity was present, as is typical in BPD samples (Table 2).

**Table 1:** Sample characteristics.

	BPD *(n = 21)*	HC *(n = 20)*	Statistic
Age (years)	27,14±7.48	24.80±5.23	T(1,39) = 1.16, p = 0.25
Education (years)	12.40±1.23	12.84±0.67	T (1,39) = 1.44, p = 0.16
EHI	28.86±7.36	30.25±6.71	T (1,39) = 0.63, p = 0.53
ALS-Total	6.52±2.23	4.16±1.76	T (1,39) = 3.75, p<0.001
BDI	14.67±10.97	1.80±2.06	T (1,39) = 5.15, p<0.001

ALS, Affective Lability Scale; BDI; EHI, Edinburgh Handedness Inventory; HC, healthy control; BPD, borderline personality disorder; S.D., standard deviation.

**Table 2:** Number of comorbid Axis I and II disorders in the BPD sample.

	n	%
**Axis I diagnoses**		
Panic disorder	15	71.4
Simple phobia	9	42.9
Generalized anxiety	2	9.5
Post-traumatic stress disorder	7	33.3
Social phobia	8	38.1
History of substance abuse/dependence	2	9.5
Major depression	10	47.6
Current major depressive episode	5	23.8
Eating disorder	9	42.9
Attention deficit hyperactivity disorder	0	0
**Axis II diagnoses**		
Paranoid	4	19.0
Schizotypal	4	19.0
Obsessive-compulsive	10	47.6
Dependent	3	14.3
Antisocial	0	0
Narcissistic	4	19.0
Avoidant	0	0
Passive-aggressive	3	14.3

### Experimental Procedure

Participants were presented with 36 black-and-white original pictures of eye gazes (12 negative, 8 positive, and 16 neutral stimuli) from the RMET paradigm, which was used successfully in prior studies (e.g., [21], [31]). All photographs were of equal size (22×8 cm). Thereafter, participants were required to choose one out of four words (three distracter words and one correct word) that describe the mental state of the person in the photograph seen before as quickly and accurately as possible. Previous studies on RMET used a simultaneous version, in which the words were displayed together with the picture [23], [32]. However, with the modified version we aimed to disentangle the neural correlates of mentalizing from those of response selection. The number of correct discriminations was calculated for all 36 items as well as for each category (neutral, negative, and positive) separately.

**Figure 1:**
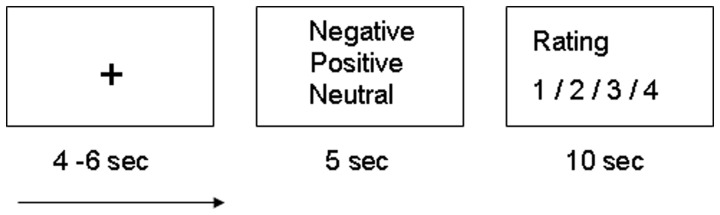
Schematic depiction of a single trial in the mental state discrimination paradigm. Each 25-s trial consisted of a 4–6 fixation cross at the beginning and at the end, a 5-s presentation of a RMET-picture (negative/positive/neutral) and a 10-s rating period. During the rating period, subjects indicated the mental state/emotion of the person on the picture (using a 4-button hand pad in their right hand). 36 trials were presented. RMET, Reading Mind in the Eyes Test (20).

Before scanning, participants were given a short practice in the scanner to become familiar with the response pad and trial structure. Then, the experimental procedure started. Each 25-s trial consisted of a 4–6 s jittered fixation cross, a 5-s presentation of a RMET picture (negative, positive or neutral), a 10-s rating period and again a 4–6-s jittered fixation cross. During the rating period, subjects indicated the mental state of the person on the picture they had seen before using a 4-button hand pad in their right hand. Each button was assigned to one of the four words, respectively. The trial structure is presented in Figure 1. All participants evaluated the same set of RMET stimuli in the same order, according to the instructions of Baron-Cohen et al. [21].

### Image Acquisition

Blood oxygenation level-dependent (BOLD) images were obtained on a 3 Tesla MRI scanner (TRIO, Siemens Medical Systems, Erlangen, Germany) system equipped with a 12 channel head coil. Changes in blood oxygenation level-dependent (BOLD) T2* weighted MR signal were measured using a gradient echo-planar imaging (EPI) sequence (TR = 2380 ms, TE = 25 ms, FoV = 210 mm, flip angle = 90°, 64 × 64 matrix, 40 slices covering the whole brain, slice thickness 3 mm, no gap, voxel size 3 × 3 × 3 mm). A T1-weighted anatomical image was additionally acquired for each subject to allow anatomical localization (TR = 2300 ms, TE = 2.98 ms, 160 slices, voxel size 1.0×1.0×1.1 mm).

### Image Processing and Statistical Analysis

Image processing and statistical analysis were conducted with Statistical Parameter Mapping [39] version 8 (Wellcome Department of Cognitive Neurology, London UK; http://www.fil.ion.ucl.ac.uk/spm). Pre-processing included realignment, co-registration, segmentation, and spatial normalization (template of Montreal Neurological Institute, MNI). Then, a Gaussian filter of 8 mm full width at half maximum was applied to smooth the data spatially. For the statistical analysis of regional differences in brain activation, positive, negative, and neutral eye gazes were put into the categorical general linear model (GLM) design at the subject level [39]. Contrasts between different conditions (Negative - Neutral, Positive - Neutral) were computed for each subject. The subtraction of the emotionally neutral condition from affective conditions is typically applied in facial emotion tasks (e.g., [40]). In the second-level analysis, one-sample *t-*tests for the contrasts negative *vs.* neutral and positive *vs.* neutral were used to obtain activation patterns for each group. In the next step, two-sample *t*-tests were analyzed to compare BPD patients and HC subjects. The probability threshold was set at *P*  = 0.001, uncorrected, for whole-brain analysis. The minimum cluster extent (*k*) was set at 10 contiguous voxels. For a priori defined regions which are implicated in mentalizing (amygdala, temporal pole, medial frontal gyrus, orbitofrontal cortex) (e.g., [24], [25]) a region of interest (ROI) approach was used with *P*<0.05, corrected for family wise errors (FWE). The regions were derived from the anatomical labelling atlas (aal) toolbox from the PickAtlas [41].

### Behavioral Data

Behavioral data (reaction time [RT], correct response rate) were analysed by means of an ANCOVA with a between-subject factor group (BPD, HC), a within-subject factor condition (negative, positive, neutral) and depression score (BDI) as a covariate.

## Results

### Behavioral Data

The main effect of BDI as the covariate was not significant in any analysis, nor was any interaction between the covariate and other factors. Therefore, depression was not included in the following results.

Both groups (HC, BPD) performed the RME task significantly better than chance (*p*<.0001). A significant main effect of group [*F*(1,40) = 7.63, *p* = 0.009] indicated that BPD patients’ correct response rate was significantly higher than that of HC subjects. More specifically, BPD patients out-performed HC in the positive as well as negative RMET condition (*p* = 0.002 and 0.007, for positive and negative items, respectively), but not in the neutral condition (*p*>0.80), resulting in a significant group × condition interaction [*F*(2,80) = 6,01, *p* = 0.005].

**Figure 2:**
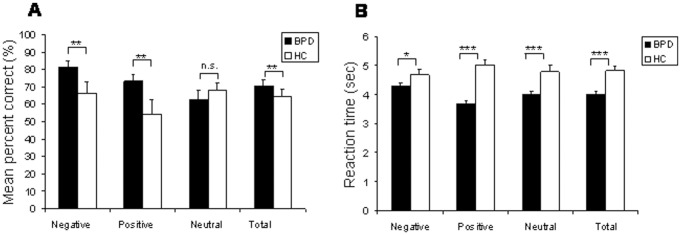
Behavioral data of the Reading the Mind in the Eyes Test (RMET). **A.** Accuracy of the RMET, **B.** Reaction Time of the RMET in borderline personality disorder (BPD) (n = 21) compared to healthy controls (HC) (n = 20). Error bars indicate standard error for mean.

As can be seen in Figure 2 (panel B), the BPD group responded significantly faster than the HC group across all conditions [*F*(1,39) = 40.44, *p*<0.001]. In addition, a significant group × condition interaction [*F*(2,78) = 18.13, *p* = 0.009] indicated that BPD patients showed fastest responses for positive eye gazes and slowest responses for negative eye gazes (positive vs. negative, *p*<.001; negative vs. neutral, *p*<.001; neutral vs. positive, *p*<.001). In contrast, HC subjects’ RT did not differ across the subscales (*p*>.80).

### Imaging Results

Brain regions demonstrating increased BOLD signal during the RMET are summarized in Table S1.

In the *negative versus neutral* contrast, HC subjects showed enhanced activation in the left insula, the temporal cortex (BA 21, 42) and the prefrontal cortex (BA 9, 11, 45). BPD patients engaged the left amygdala, the temporal cortex (BA 21, 38), the left prefrontal cortex (BA 10, 11, 32), the right hippocampus, the left parietal cortex (BA 31, 40) and the left occipital cortex (BA 18).

In the *positive versus neutral* contrast, HC subjects showed larger activation in the temporal cortex (BA 38), the prefrontal cortes (BA 9, 10), the right thalamus and the right occipital cortex (BA 18). BPD patients showed enhanced activation in the left temporal cortex (BA 21), the prefrontal cortex (BA 10, 47) and the occipital cortex (BA 18).

### Group Comparisons

**Figure 3:**
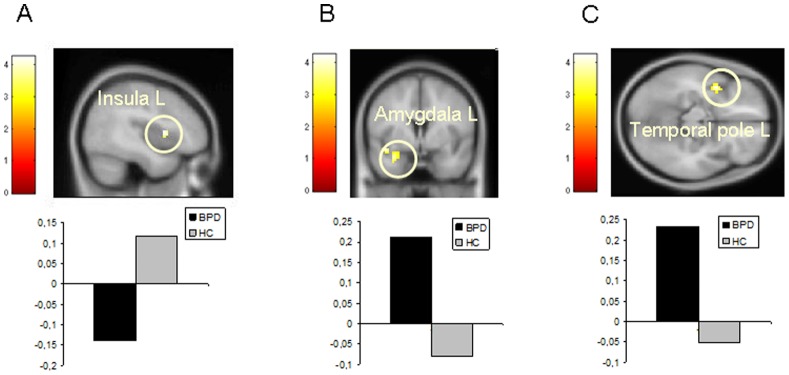
Group comparisons during negative *versus* neutral eye gazes. The color bar indicates t values. The display threshold is p<0.05 (FWE-corrected). FMRI images comparing **A.** HC to BPD; **B.** BPD to HC; **C.** BPD to HC.

A between group comparison in the *negative* condition demonstrated a greater activation of the right inferior frontal gyrus (BA 45), the right insula (BA 13) and the right superior temporal pole (BA 22) in HC subjects than in BPD patients. In contrast, the activity in the left amygdala, the left temporal pole (BA 38), the medial frontal gyrus (BA 6, 9), the right middle temporal gyrus (BA21), the left precuneus (BA 31) and the left middle occipital gyrus (BA18) was larger in BPD than in HC (see Table 3 and Figure 3).

**Table 3:** Significant differences in BOLD signal between groups during the RMET.

MNI coordinates					
Region	k	x	y	z	*T*
**HC > BPD**					
*Negative > Neutral*					
R inferior frontal gyrus (BA45)	39	45	29	1	4.82
R insula (BA13)	10	42	8	4	4.48
R superior temporal pole (BA22)	10	60	11	−5	3.85*
*Positive > Neutral*					
L parietal lobe (BA7)	232	−24	−46	58	4.95
R posterior cingulate gyrus (BA29)	11	9	−43	19	4.92
R superior temporal gyrus (BA22)	86	33	−55	16	4.91*
R hippocampus	14	30	−19	−17	4.85
L postcentral gyrus (BA4)	24	−48	−16	40	4.60
R insula (BA13)	155	39	−1	−2	5.36
L insula (BA13)	45	−36	−19	7	5.07
R medial frontal gyrus (BA6)	121	3	11	49	4.45*
R superior temporal pole (BA22)	33	57	11	−2	5.18*
R middle temporal gyrus/posterior STS (BA37)	41	57	−67	7	4.31*
**BPD > HC**					
*Negative > Neutral*					
L precuneus (BA31)	16	−3	−61	22	3.88
L temporal pole (BA38)	63	−42	5	−14	4.26*
L middle occipital gyrus (BA18)	16	−24	−97	13	3.90
L amygdala (BA28)	10	−30	2	−23	3.61*
R medial frontal gyrus (BA6)	12	12	−16	52	3.40*
L fronto superior medial gyrus (BA9)	12	−12	62	31	3.34*
R middle temporal gyrus (BA21)	16	57	−13	−11	3.58*
*Positive > Neutral*					
L inferior frontal orbital gyrus (BA47)	20	−42	29	−8	4.45
R middle temporal gyrus (BA41)	17	48	−40	13	4.11*
L superior temporal gyrus (BA40)	11	−51	−46	22	3.85*
R medial frontal gyrus (BA10)	21	12	50	13	3.79*
L medial frontal gyrus (BA8)	13	−12	41	34	3.17*
L temporal pole (BA38)	12	−45	14	−23	3.78*
L middle temporal gyrus (BA21)	14	−51	−1	−23	3.25*
R amygdala (BA36)	9	33	−1	−29	3.59*

Notes: k =  cluster size in voxels. All comparisons are significant at *p*<0.001 (uncorrected),

* p<.05 (FWE-corrected); minimum k = 10.

BA, Brodmann area; L, left; MNI, Montreal Neurological Institute; R, right.

**Figure 4:**
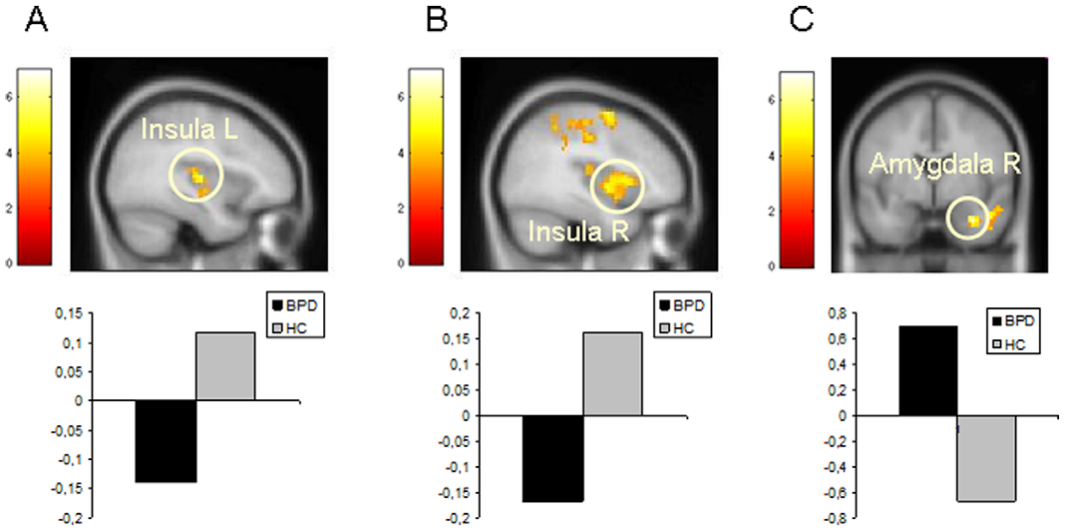
Group comparisons during positive *versus* neutral eye gazes. The color bar indicates t values. The display threshold is p<0.05 (FWE-corrected). FMRI images comparing **A.** HC to BPD; **B.** BPD to HC; **C.** BPD to HC.

In the *positive* condition, the activation in the insula (BA 13), the right medial frontal gyrus (BA 6), the right superior temporal pole (BA 22), the left parietal lobe (BA 7), the right posterior cingulate gyrus (BA 29), the left postcentral gyrus (BA 4), the right middle temporal gyrus (BA 37), and the right hippocampus was larger in HC than in BPD patients. In contrast, BPD patients engaged the right amygdala, the left orbitofrontal gyrus (BA 47), the medial frontal gyrus (BA 8, 10), the right middle temporal gyrus (BA 41), the left superior temporal gyrus (BA 40), the left temporal pole (BA 38), and the left middle temporal gyrus (BA 21) to a larger extent than HC subjects (see Table 3 and Figure 4).

## Discussion

To our best knowledge, this is the first study examining neural underpinnings of mentalizing abilities in BPD patients using the RMET.

### Behavioral Data

In line with Fertuck et al. [30], BPD patients demonstrated superior mental state discrimination than healthy controls, manifested in a significant main group effect. However, this effect resulted from the patients’ superior mental state discrimination of positive and negative eye gazes, whereas no group differences were found for neutral eye gazes. In contrast to Fertuck et al. [30], our results were independent of depression severity, which might be explained by the fact that our sample contained outpatients with moderate levels of depression. Apparently, moderate levels of depression are not responsible for better mental state discrimination in BPD. Our findings of superior mindreading abilities are also consistent with the study of Scott et al. [31], who reported enhanced accuracy of negative eye gaze discrimination in healthy individuals with high- versus low subclinical borderline features. In contrast to that study, however, in this current study BPD patients showed significantly faster responses regardless of stimulus valence. Likewise, Lynch et al. [18] using a facial morphing procedure found enhanced emotion recognition (i.e. higher correct response rates) and heightened sensitivity (i.e., faster response rates) in BPD. The faster responses in BPD patients might reflect judgements on a more intuitive level than in healthy subjects.

The finding of superior mentalizing abilities in BPD stands in contrast to the finding of Preißler et al. [32], who did not report differences in mentalizing between BPD and HC subjects Likewise, a recent study on RMET with a larger sample (n = 31) did not find group differences between BPD patients and healthy subjects [42]. Moreover, the authors did not report group differences in BPD patients with and without depression or anxiety. However, in both studies, some patients were medicated with psychotropic medications (e.g., antidepressants), which may have influenced the results. In addition, our results contradict other studies using facial recognition tasks, which even reported impaired emotion recognition in BPD patients as compared to HC subjects [13], [43]. To sum up, the results of the present study support the paradox hypothesis of Krohn [11] which assumes that BPD patients have enhanced mentalizing abilities in spite of problems in social relationships. This finding seems to be unrelated to the very limited amount of stimulation in the RMET as compared to facial tasks. The divergent findings might be the consequence of depression severity, patient sample (inpatients vs. outpatients) and medication.

### Neural Responses in BPD and HC Subjects

In accordance with prior studies on mental state discrimination [22], [24], brain activity in healthy subjects during affective stimuli was located in the middle temporal gyrus (BA 21), the left temporal poles (BA 22) and the prefrontal cortex. Additionally, negative eye gazes elicited a greater activity in the left orbitofrontal cortex and left insula and positive eye gazes elicited a greater activity in the thalamus and the occipital cortex. In contrast to Baron-Cohen et al. [24], no amygdala activation was found in HC. BPD patients showed a greater activity during negative and positive eye gazes in the amygdala, the middle temporal gyrus (BA 21), the right temporal poles (BA 38) and the orbitofrontal cortex (BA 11, 47). In addition, a number of regions in patients responded specifically to negative eye gazes: the right hippocampus, the left temporal pole, the ACC (BA 32), the medial PFC (BA 10), the left inferior parietal gyrus (BA 40) and the left precuneus (BA 31).

In line with our hypothesis, group comparison demonstrated enhanced amygdalar activation during negative (left amygdala) and positive (right amygdala) mental state discrimination in BPD patients compared to healthy subjects. Likewise, two studies using facial emotion recognition tasks found a larger amygdala activity in BPD than in HC [15], [44], though this finding was not replicated in a third study [20]. The amygdala plays a key role in automatic non-conscious processing of emotions, in processing emotionally arousing stimuli, both pleasant and aversive, as well as in the monitoring of eye gazes [45]. It has also been suggested that the amygdala is involved in the allocation of resources to process various kinds of biologically salient stimuli [46], [47].

In addition, BPD patients responded to both positive and negative stimuli with a larger activation in the left temporal pole (BA 38) and the middle temporal gyrus (BA 21) compared to HC subjects. The temporal pole is regarded as a higher-order visual cortical area, highly interconnected with both the amygdala and the prefrontal cortex [48]. The temporal pole binds complex, highly processed perceptual inputs to visceral emotional responses and plays a key role in face processing and ToM [24], [49], [50]. The additional activation of the temporal pole and medial frontal gyrus in BPD subjects compared with HC may, therefore, be interpreted as an index of their more profound emotional processing of visual stimuli. The left inferior frontal gyrus (BA 45) is a pre-motor area believed to be part of the so-called ‘mirror’ neuron system that is activated by both movement observation and execution. This mirror motor representation is suggested to underlie the understanding of motor events, thus making communication and mind reading possible [48]. In sum, the data indicate that BPD patients not only can better estimate the emotional state of the other on the basis of eye gaze, but also “resonate” with the other person’s mental state in their own emotional response. In turn, healthy subjects showed stronger activation than BPD patients in the bilateral insula and the right STG (to all affective stimuli), in the right inferior frontal gyrus (to negative stimuli), as well as in the left primary somatosensory cortex, the left parietal lobe, the right STS, the PCC and the right hippocampus (to positive stimuli). All these areas, except hippocampus, are typically activated in experiments for empathy (e.g., [51], [52]).

The findings fit well with the assumption of Fonagy and Bateman that in BPD the ability to mentalize is developed only partially, based on traumatic experiences and reduced mirroring of the emotional state of the child by the parents [53], [54]. According to Ghiassi et al. [55], healthy subjects learn to understand their own feelings and predict actions of their caregivers by a mirroring process. In contrast, BPD patients who grow up in a non-validating environment might have developed the ability to a more intuitive emotional evaluation without reflexive awareness. These different strategies might be reflected in the activation pattern characterized by an enhanced amygdala activity and reduced activation in brain regions associated with the mirror neuron system as seen in the present study. Thus, a putative interpretation of these data might be that while BPD patients intuitively and automatically “resonate” with mental states of others, HC subjects recruit brain areas associated with conscious emotional representation of the mental states of others. That is, BPD patients might have an overactive and exaggerated resonance with other’s mental state with weaker top-down modulation.

### Limitations

In this first study of the neural underpinnings of mental state discrimination in borderline patients using RMET, several limitations should be mentioned: first, our sample comprised seven BPD patients with comorbid PTSD. Although we did not find differences between BPD patients with and without PTSD, this effect cannot be ruled out completely. Additionally, we cannot rule out that other comorbid disorders, such as panic disorder, eating disorder or social phobia have a relevant impact on our results. On the other side, comorbid disorders represent the typical clinical picture of borderline patients [56]. Thus, exclusion would have led to a sample of a non-representative patient group. The study by Fertuck et al. [30] on a slightly larger sample (n = 25) found that the impact of depressive symptoms (which were controlled in the present study) to the RMET findings was much larger than the non-significant impact of SCID I and II disorders. Nevertheless, future studies should better control SCID I and II disorders as well. Second, the RMET assesses only subtle mental states. Thus, no statements are possible regarding more complex scenes of social interactions. Future studies should include more complex mentalizing tasks to establish whether enhanced mentalizing is restricted to simple stimuli, such as the eye region, or whether superior mentalizing is a general phenomenon in BPD.

### Conclusions

In sum, our neurophysiological findings are in accordance with the view that BPD patients are highly vigilant of social stimuli [57]. Our results indicate that BPD patients are better in the attribution of mental states on the basis of very limited information than healthy controls, thus supporting the position of Krohn [11], who labeled the apparent contradiction between the impaired interpersonal relations and the enhanced emotional sensitivity as a ‘paradox’ specific to borderline psychopathology.

## Supporting Information

Table S1 Notes: k = cluster size voxels. *p*<0.001 (uncorrected), *p<.05 (FWE-corrected); minimum cluster size, k = 10. BA, broadman area; L, left; MNI, Montreal Neurological Institute; R, right.
(DOC)
