# Supplementary material for: Hypersensitivity in Borderline Personality Disorder during Mindreading
Source: PLoS One. 2012 Aug 3;7(8):e41650. doi: 10.1371/journal.pone.0041650 (PMC3411703; doi:10.1371/journal.pone.0041650)
Supplement: Table S1 — Notes: k = cluster size voxels. p<0.001 (uncorrected), *p<.05 (FWE-corrected); minimum cluster size, k = 10. BA, broadman area; L, left; MNI, Montreal Neurological Institute; R, right. (DOC) [file pone.0041650.s001.doc]

**Supplementary S1.** Brain ativation in healthy control subjects (HC) during the RMET. MNI, Montreal Neurological Institute.

| MNI coordinates | | | | | |
| --- | --- | --- | --- | --- | --- |
| **Region** | **k** | **x** | **y** | **z** | **T** |
|  |  |  |  |  |  |
| **Healthy Controls** |  |  |  |  |  |
|  |  |  |  |  |  |
| *Negative vs. Neutral* |  |  |  |  |  |
| L inferior frontal gyrus tri (BA45) | 378 | -39 | 23 | 7 | 11.53 |
| L middle temporal gyrus (BA21) | 27 | -54 | 2 | -23 | 8.04 |
| R middle frontal gyrus (BA6) | 63 | 48 | 5 | 52 | 6.55 |
| L medial orbitofrontal gyrus (BA11) | 23 | -6 | 44 | -14 | 6.20 |
| L superior frontal gyrus (BA6) | 146 | 9 | 11 | 64 | 5.98 |
| L middle frontal gyrus (BA6) | 19 | -51 | 11 | 43 | 5.97 |
| L insula (BA13) | 41 | -39 | -16 | 10 | 4.96 |
| L superior frontal gyrus (BA9) | 26 | -12 | 56 | 28 | 5.68 |
| L middle frontal gyrus (BA9) | 25 | -39 | 44 | 28 | 5.58 |
| R superior temporal gyrus (BA42) | 19 | 66 | -28 | 10 | 5.48 |
|  |  |  |  |  |  |
| *Positive > Neutral* |  |  |  |  |  |
| R occipital inferior gyrus (BA18) | 449 | 33 | -85 | -11 | 8.35 |
| R thalamus | 188 | 18 | -13 | 7 | 7.66 |
| L middle frontal gyrus (BA9) | 26 | -36 | 41 | 31 | 5.32 |
| R middle frontal gyrus (BA10) | 80 | 36 | 53 | 19 | 5.22 |
| L superior frontal gyrus (BA6) | 45 | -21 | 2 | 70 | 5.12 |
| L medial frontal gyrus (BA6) | 177 | -6 | -1 | 67 | 6.14 |
| L superior temporal pole (BA38) | 20 | -57 | 5 | -8 | 4.03 |
| R superior temporal pole (BA38) | 17 | 57 | 14 | -5 | 3.44 |
|  |  |  |  |  |  |
| **BPD patients** |  |  |  |  |  |
|  |  |  |  |  |  |
| *Negative > Neutral* |  |  |  |  |  |
| L middle temporal gyrus (BA21) | 36 | -63 | -10 | -14 | 9.29 |
| L occipital gyrus (BA18) | 84 | -15 | -97 | 19 | 6.94 |
| R hippocampus (BA41) | 25 | 36 | -34 | 7 | 5.15 |
| L temporal pole (BA38) | 72 | -42 | 2 | -17 | 4.75 |
| L anterior cingulate gyrus (BA32) | 27 | 0 | 23 | -8 | 4.37 |
| R middle temporal gyrus (BA21) | 14 | 45 | 5 | -26 | 4.57 |
| R cuneus (BA18) | 66 | 12 | -85 | 19 | 4.45 |
| L precuneus (BA31) | 16 | -3 | -61 | 22 | 4.06 |
| L inferior parietal gyrus (BA40) | 18 | -57 | -31 | 25 | 4.02 |
| L amygdala | 18 | -27 | -1 | -20 | 4.38 |
| L superior frontomedial gyrus (BA10) | 22 | -12 | 65 | 28 | 4.48 |
| L medial frontal gyrus (BA11) | 42 | -3 | 38 | -17 | 3.94 |
| L fronto middle orbital gyrus (BA11) | 41 | -3 | 50 | -14 | 3.64 |
|  |  |  |  |  |  |
| *Positive > Neutral* |  |  |  |  |  |
| L middle temporal gyrus (BA21) | 66 | -51 | 2 | -20 | 7.63 |
| L inferior orbital gyrus (BA47) | 23 | -39 | 29 | -8 | 5.87 |
| R cuneus (BA18) | 125 | 12 | -82 | 19 | 5.78 |
| L middle occipital gyrus (BA18) | 44 | -21 | -94 | 13 | 5.22 |
| R fronto superior medial gyrus (BA10) | 23 | 9 | 68 | 25 | 4.75 |
| L superior temporal pole (BA21) | 28 | -51 | 5 | -20 | 6.46 |
